# Deep learning models for interpretation of point of care ultrasound in military working dogs

**DOI:** 10.3389/fvets.2024.1374890

**Published:** 2024-06-06

**Authors:** Sofia I. Hernandez Torres, Lawrence Holland, Thomas H. Edwards, Emilee C. Venn, Eric J. Snider

**Affiliations:** ^1^Organ Support and Automation Technologies Group, U.S. Army Institute of Surgical Research, JBSA Fort Sam Houston, San Antonio, TX, United States; ^2^Hemorrhage Control and Vascular Dysfunction Group, U.S. Army Institute of Surgical Research, JBSA Fort Sam Houston, San Antonio, TX, United States; ^3^Texas A&M University, School of Veterinary Medicine, College Station, TX, United States; ^4^Veterinary Support Group, U.S. Army Institute of Surgical Research, JBSA Fort Sam Houston, San Antonio, TX, United States

**Keywords:** ultrasound imaging, military medicine, canine, deep learning, triage, abdominal hemorrhage, pneumothorax, hemothorax

## Abstract

**Introduction:**

Military working dogs (MWDs) are essential for military operations in a wide range of missions. With this pivotal role, MWDs can become casualties requiring specialized veterinary care that may not always be available far forward on the battlefield. Some injuries such as pneumothorax, hemothorax, or abdominal hemorrhage can be diagnosed using point of care ultrasound (POCUS) such as the Global FAST® exam. This presents a unique opportunity for artificial intelligence (AI) to aid in the interpretation of ultrasound images. In this article, deep learning classification neural networks were developed for POCUS assessment in MWDs.

**Methods:**

Images were collected in five MWDs under general anesthesia or deep sedation for all scan points in the Global FAST® exam. For representative injuries, a cadaver model was used from which positive and negative injury images were captured. A total of 327 ultrasound clips were captured and split across scan points for training three different AI network architectures: MobileNetV2, DarkNet-19, and ShrapML. Gradient class activation mapping (GradCAM) overlays were generated for representative images to better explain AI predictions.

**Results:**

Performance of AI models reached over 82% accuracy for all scan points. The model with the highest performance was trained with the MobileNetV2 network for the cystocolic scan point achieving 99.8% accuracy. Across all trained networks the diaphragmatic hepatorenal scan point had the best overall performance. However, GradCAM overlays showed that the models with highest accuracy, like MobileNetV2, were not always identifying relevant features. Conversely, the GradCAM heatmaps for ShrapML show general agreement with regions most indicative of fluid accumulation.

**Discussion:**

Overall, the AI models developed can automate POCUS predictions in MWDs. Preliminarily, ShrapML had the strongest performance and prediction rate paired with accurately tracking fluid accumulation sites, making it the most suitable option for eventual real-time deployment with ultrasound systems. Further integration of this technology with imaging technologies will expand use of POCUS-based triage of MWDs.

## 1 Introduction

Ultrasound is commonly used in canines with suspected abdominal or thoracic injuries following trauma, to identify free fluid which may require surgical intervention. Different standardized exams are used in veterinary medicine such as the abdominal focused assessment with sonography for trauma (AFAST®), thoracic FAST (TFAST®), or the Veterinary Bedside Lung Ultrasound Exam (Vet BLUE®) (1–3). These are often performed together and referred to as GlobalFAST® which can be used for civilian trauma cases, but also for working dog casualties (4). Working dogs cover a wide range of occupations including military working dogs (MWDs) which go anywhere soldiers are deployed and aid with a wide range of tasks (5). The ever increasing high risk mission that MWDs share with their handlers puts them at risk for similar injuries as their Service member counterparts (6, 7). Unfortunately, in the early roles of care, where MWD casualties are first managed, veterinary expertise may not be present to properly acquire ultrasound images and to interpret images making GlobalFAST® inaccessible for treatment of MWDs at these early stages of care (8).

This is further complicated on the future battlefield where medical evacuation will be limited and more medical care and triage will need to be provided in theater, at early roles of care (9). In fact, this is already being experienced with the Ukraine-Russia conflict, where limited medical evacuation opportunities arise due to challenged airspace, which is requiring far forward surgical teams to treat and manage a larger number of casualties for up to 72 h in theater (10). This is further complicated by precise long-range weaponry minimizing the relative safety of CASEVAC even at distances above 500 km away from enemy lines. In addition, more than 70% of Ukraine casualties stem from more advanced rocket or artillery injuries, which often result in complex polytrauma to multiple organ systems (10). Thus, as we look towards the future battlefield, it is even more imperative to have accurate triage procedures for prioritizing injured warfighters for access to limited evacuation opportunities.

Towards addressing this critical capability gap for canine and human casualties on the future battlefield, artificial intelligence (AI) can be utilized to automate medical triage image interpretation (11, 12). AI for image interpretation often relies on deep convolutional neural network models containing millions of trainable parameters to extract features from images for making categorical predictions (13, 14). For medical applications, AI has been widely used for tumor detection (15, 16), COVID-19 diagnosis (17, 18), and obstetric ultrasound applications (19, 20). In addition, AI has been applied to interpret radiographs in thoracic (21, 22), cardiac (23, 24), and orthopedic (25) settings. Our research team has previously developed an ultrasound image AI interpretation model for detecting shrapnel in tissue, termed ShrapML (26, 27). We have recently expanded this work to the enhanced FAST (eFAST) exam commonly used for human emergency triage applications (28). This application resulted in different AI models for detecting pneumothorax, hemothorax, and abdominal hemorrhage injuries in tissue phantom image sets. In this presented work, we hypothesize if AI image interpretation models are trained on canine image datasets, they will be able to automatically identify injuries at each POCUS scan point. By doing so, the skill threshold for POCUS interpretation will be lowered so that this critical triage task can be available at early echelons of care where emergency intervention is most needed for MWDs.

## 2 Materials and methods

### 2.1 Imaging protocol

Research was conducted in compliance with the Animal Welfare Act, implementing Animal Welfare regulations, and the principles of the Guide for the Care and Use for Laboratory Animals. The Institutional Animal Care and Use Committee at the Department of Defense Military Working Dog Veterinary Services approved all research conducted in this study. The facility where this research was conducted is fully accredited by the AAALAC International. The POCUS protocol used mirrored the GlobalFAST® procedure in a total of five (1.5 to 10 years old) healthy canine subjects (20 to 55 kgs weight) under general anesthesia or deep sedation for other medical procedures, as prescribed by the attending veterinarian. Ultrasound (US) clips were collected in 8 scan points (Table 1) using a C11 transducer (Fujifilm, Bothell, WA, United States) with a Sonosite Edge ultrasound system (Fujifilm, Bothell, WA, United States). The subject was positioned in right lateral, left lateral, sternal or dorsal recumbency for ease of access to each scan point. A minimum of three 15 s clips were collected at each scan point with the probe orientation held in the coronal plane for the first 6 s and then rotated to the transverse plane for the remainder of each clip. All clips collected from the live subjects were used as baseline (negative for injury) data. The same scanning protocol was used to obtain US imaging data from a cadaver canine model. A total of five frozen cadavers (Skulls Unlimited, Oklahoma City, OK, United States) were received and stored at −20°C until ready for use. Once thawed, an endotracheal tube (McKesson Medical-Surgical, Irving, TX, United States) was placed into the trachea of each subject and secured to a bag valve mask (EMS Safety Services, Eugene, OR, United States) for ventilation. At this time thoracic and abdominal CT scans (Toshiba Aquilion CT Scanner, Cannon Medical Systems, Tustin, CA, United States) were collected to identify any pre-existing injuries. Then, data was collected at each scan point, using the same protocol as the live subjects. After collecting the first round of data, if the subject was positive for any injury, e.g., a pneumothorax, a needle decompression was performed to remove air and obtain a negative scan. Another round of data was collected with the scan points that were negative for injury. Next, controlled injuries were performed by adding blood or saline to the pleural space (up to 300 mL) or the abdomen (up to 400 mL) for a final round of positive injury image collection in the cadaver subjects.

**Table 1:** Scan point description for the POCUS imaging protocol.

Scan point	Abbreviation	Description
Bilateral Chest Tube Site	CTS	Longitudinal plane on both sides of the chest perpendicular to the ribs at the 7th to 9th intercostal space.
Bilateral Pericardial Site	PCS	Longitudinal and transverse planes on each side of the chest between the 5th and 6th intercostal spaces over the heart.
Diaphragmatic Hepatic	DH	Subxiphoid view for visualization of the pleural and pericardial spaces beyond the diaphragm to evaluate hepatodiaphragmatic interface, gallbladder region, and pericardial sac.
Splenorenal	SR	Left flank view to assess the splenorenal interface and areas between the spleen and body wall
Cystocolic	CC	Midline view to assess the apex of the bladder
Hepatorenal	HR	Right flank view to assess the hepatorenal interface and areas between the spleen and body wall

### 2.2 Preprocessing images

All clips were exported from the US machine as MP4 format and then renamed to reflect the scan point, subject ID, and recumbency of each subject. Frames were extracted from each clip using ffmpeg tool, via a Ruby script, and then sorted by positive or negative for injury by scan point. Each frame was then cropped to remove the user interface information from the US system and the images were resized to 512 × 512 pixels. Additional steps were taken with images collected at the chest tube site, to recreate M-mode images. Briefly, clips were processed to extract a pixel-wide image over time for visualizing the lung-pleura interface movement. These custom-M-mode images were then cropped and resized to 512 × 512 as well.

Before images were ready for training, they were augmented to prevent model overfitting and improve performance. While data augmentation is useful to prevent overfitting, it can result in poor model performance and more computationally intensive training if not setup optimally for the application (29). A representative image was chosen from each scan point, including M-mode reconstructions, to match histogram values across all the other images using “imhistmatch” function by MATLAB (MathWorks, Natick, MA, United States). Then, contrast and brightness were randomly adjusted by ±20% to add training noise using the “jitterColorHSV” function by MATLAB. Both MATLAB functions were applied to all images for every scan point using Image Batch Processor on MATLAB. Augmented US images were imported at a 512 × 512 × 3 image size and were randomly assigned to training, validation or testing datasets at a 70:15:15 ratio. Image sets were set up so that an even number of positive or negative images were selected in each dataset for each split. Next, training images were augmented randomly by affine transformations: random scaling, random X and Y reflections, random rotation, random X and Y shear, and random X and Y translation. However, for the CTS M-mode scan point only X reflection and translation affine transformations were applied given how these images were constructed. Due to DH scan point images being unable to train with all augmentations (data not shown), only reflection and translation augmentations were applied for both the X and Y direction.

### 2.3 Training AI models

Three different AI models were evaluated for this application that have previously been used for ultrasound image interpretation successfully – MobileNetV2 (30), DarkNet-19 (31), and ShrapML (26). MobileNetV2 has 53 convolutional layers, 3.5 million parameters, and was optimized for use on mobile devices. We have previously shown this architecture to perform at the highest accuracy for identifying shrapnel in a custom tissue phantom. The second-best performing architecture, DarkNet-19, has 19 convolutional layers, 20.8 million parameters, and utilizes global average pooling for making predictions. The last model used, ShrapML, was purpose built and Bayesian optimized for identifying shrapnel in ultrasound images at a high accuracy and much more rapid than conventional models. In addition, we have shown it to be successful at identifying pneumothorax, hemothorax, and abdominal hemorrhage injuries in eFAST images captured in human tissue phantom models (28). ShrapML consists of 8 convolutional layers with only 430,000 trainable parameters.

Training for all scan points consisted of a learning rate of 0.001 with a batch size of 32 images and RMSprop (root mean squared propagation) as the optimizer. A maximum of 100 epochs was allowed for training with a validation patience of 5 epochs if the overall validation loss did not improve. The model with the lowest validation loss was selected for use with blind predictions. All training was performed using MATLAB R2022b run on a Microsoft Windows workstation with a NVIDIA GeForce RTX 3090 Ti 24Gb VRAM graphics card, Intel i9-12900k and 64 GB RAM.

### 2.4 Performance metrics

Testing image sets were used to assess blind performance in multiple ways. First, confusion matrices were generated to categorize prediction as either true positive (TP), true negative (TN), false positive (FP), or false negative (FN) results. These results were used to generate performance metrics for accuracy Eq. 1, precision Eq. 2, recall Eq. 3, specificity Eq. 4, and F1 scores Eq. 5 using commonly used formulas for each.


(1) $$\mathrm{Accuracy}=\frac{\left(TP+TN\right)}{\left(TP+TN+FP+FN\right)}$$

(2) $$\mathrm{Precision}=\frac{TP}{TP+FP}$$

(3) $$\mathrm{Recall}=\frac{TP}{TP+FN}$$

(4) $$\mathrm{Specificity}=\frac{TN}{TN+FP}$$

(5) $$F1\;\mathrm{score}=\frac{2\times \mathrm{Precision}\times \mathrm{Recall}}{\mathrm{Precision}+\mathrm{Recall}}$$

Then, we constructed receiver operating characteristic (ROC) plots to further classify performance for a number of confidence thresholds for the predictions. ROC plots were used to calculate the area under the ROC curve or AUROC, which tells you how well the model differentiates between categories. Next, inference time for test image predictions were quantified for each trained model to assess differences in computational efficiency of the three different AI models used. Lastly, Gradient-weighted Class Activation Mapping (GradCAM) overlays were generated for test predictions to highlight the regions of images where the AI predictions were focused (32). These were used as an explainable-AI methodology to verify the AI models were accurately tracking the image regions where injury differences were present (16, 33, 34).

## 3 Results

### 3.1 MobileNetV2

**Figure 1:**
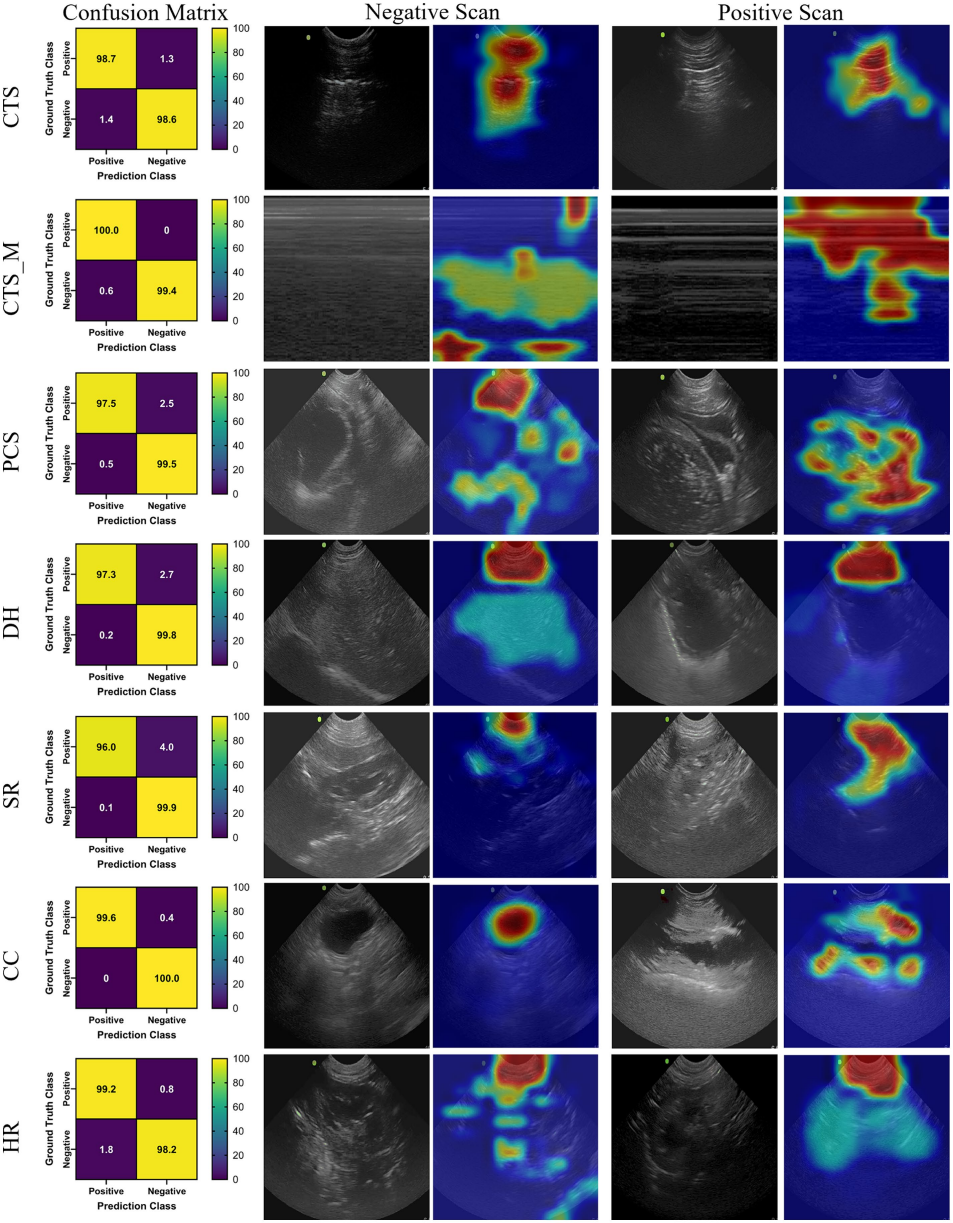
Prediction results by scan point for MobileNetV2. Results for each scan site showing (column 1) confusion matrix test prediction results, (column 2–3) negative and (column 4–5) positive representative images without and with the GradCAM overlay. Regions in the images with high relevance to model predictions have red-yellow overlays, while those of lower relevance have blue-green overlays.

MobileNetV2 was successfully trained for each POCUS scan point, with an average accuracy across all locations of 98.8% (Table 2). In addition, strong performance was evident for other conventional metrics across each POCUS scan point. However, upon closer inspection using GradCAM mask overlays, the MobileNetV2 trained model was not always properly tracking the injury site, but instead was focused on image artifacts that will likely not be consistent for additional canine subjects not included in the current datasets (Figure 1). CTS scan sites for both M- and B-mode were accurately tracking injuries, other scan sites such as HR, DH, and SR were not tracking correctly. Average inference times across all MobileNetV2 scan site models was 6.21 ms per prediction.

**Table 2:** Summary of performance metrics for MobileNetV2.

Metric	CTS	CTS M-mode	PCS	DH	SR	CC	HR	Average
Accuracy	0.987	0.997	0.985	0.986	0.979	0.998	0.987	0.988
Precision	0.986	0.994	0.995	0.998	0.999	1.000	0.982	0.995
Recall	0.987	1.000	0.976	0.973	0.960	0.996	0.992	0.980
Specificity	0.986	0.994	0.995	0.998	0.999	1.000	0.982	0.995
F1 Score	0.987	0.997	0.985	0.985	0.979	0.998	0.987	0.987
AUROC	0.999	1.000	1.000	1.000	0.999	1.000	0.999	1.000
Inference Time (ms/image)	6.22	7.67	5.59	5.58	6.64	6.06	6.57	6.21

### 3.2 DarkNet-19

**Figure 2:**
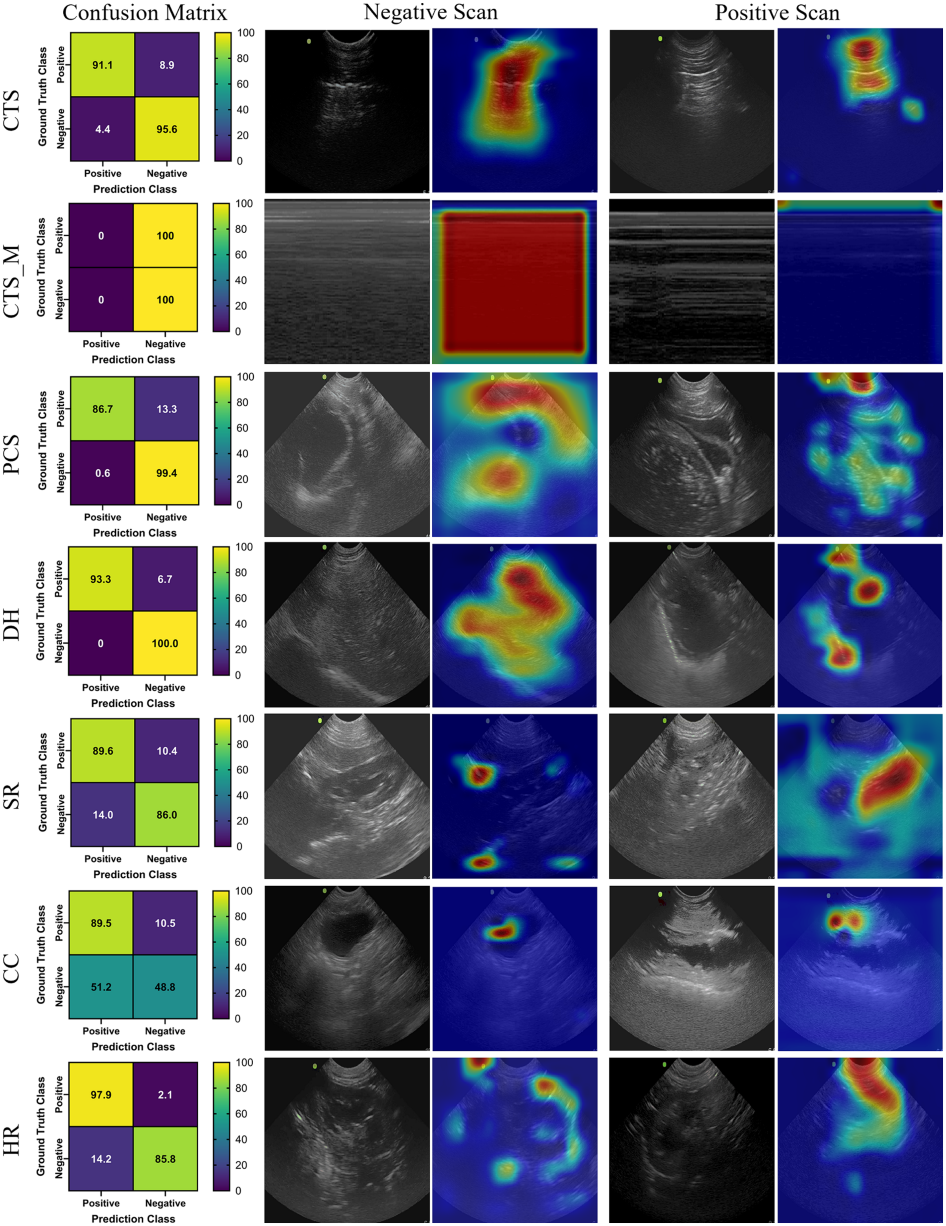
Prediction results by scan point for DarkNet-19. Results for each scan site showing (column 1) confusion matrix test prediction results, (column 2–3) negative and (column 4–5) positive representative images without and with the GradCAM overlay. Regions in the images with high relevance to model predictions have red-yellow overlays, while those of lower relevance have blue-green overlays.

The DarkNet-19 models had similar inference speeds compared to MobileNetV2 at 5.93 ms per prediction, but overall performance was reduced for a number of the scan sites, resulting in an average accuracy across all scan points of 86.4% (Table 3). Certain scan points like chest-tube M-mode images resulted only in predictions of negative (TN or FN) and the GradCAM overlays identified no obvious tracked features in the image (Figure 2). While this was the worst performing dataset trained against, the Cystocolic scan site was also only at 69.2% accuracy. While performance was reduced compared to MobileNetV2 across nearly all metrics, the GradCAM overlays were more accurately tracking image features consistent with locations where free fluid was or could be identified. These results indicated that while performance was overall reduced for DarkNet-19, the predictions were more often tracking the proper image features. More images and subject variability may improve on training performance.

**Table 3:** Summary of performance metrics for DarkNet-19.

Metric	CTS	CTS M-mode	PCS	DH	SR	CC	HR	Average
Accuracy	0.933	0.500	0.930	0.967	0.878	0.692	0.919	0.864
Precision	0.954		0.993	1.000	0.865	0.636	0.873	0.844
Recall	0.911	0.000	0.867	0.933	0.896	0.895	0.979	0.926
Specificity	0.956	1.000	0.994	1.000	0.860	0.488	0.858	0.801
F1 Score	0.932		0.926	0.966	0.880	0.744	0.923	0.878
AUROC	0.984	0.575	0.992	0.999	0.953	0.737	0.988	0.92
Inference Time (ms/image)	6.32	8.73	5.53	5.61	5.86	6.17	6.07	5.93

### 3.3 ShrapML

**Figure 3:**
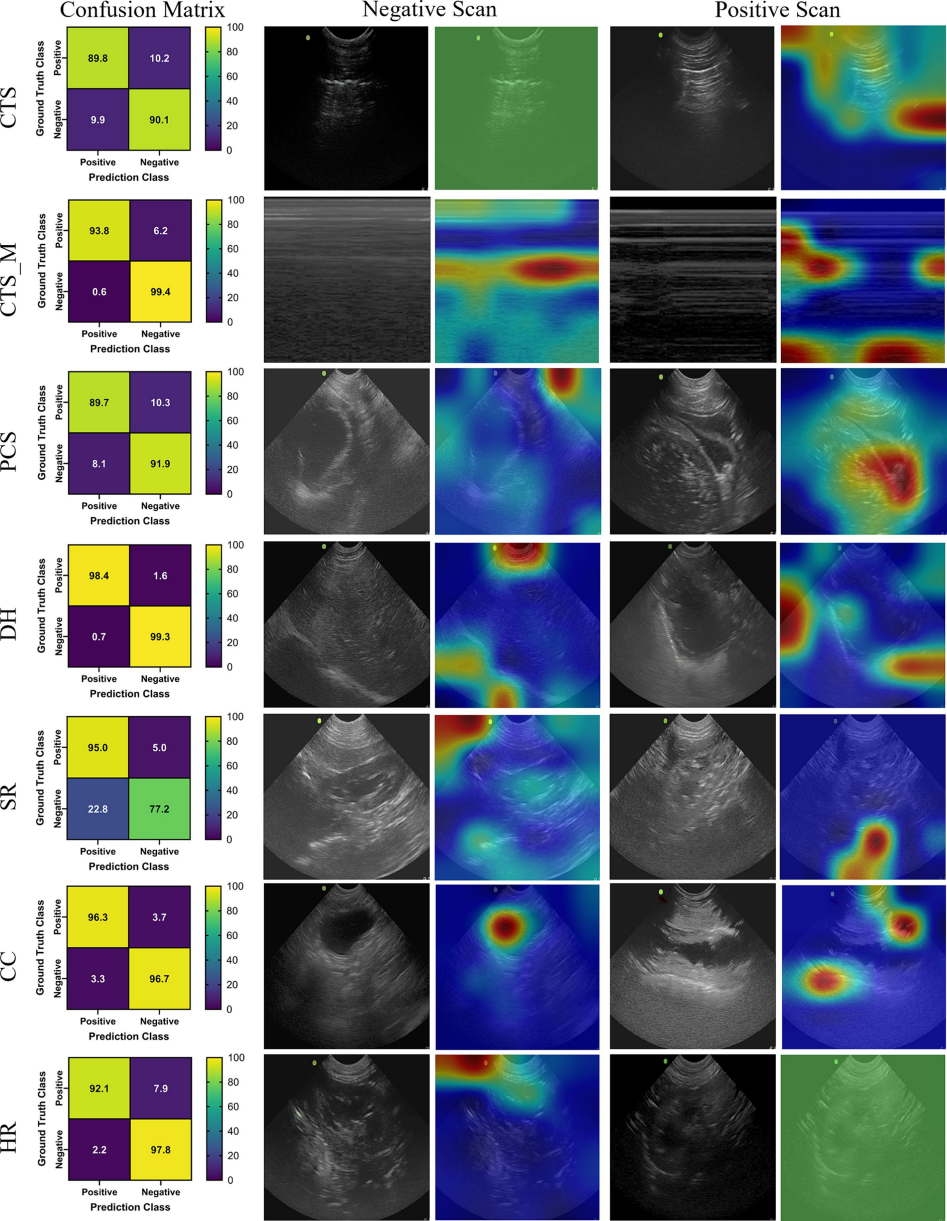
Prediction results by scan point for ShrapML. Results for each scan site showing (column 1) confusion matrix test prediction results, (column 2–3) negative and (column 4–5) positive representative images without and with the GradCAM overlay. Regions in the images with high relevance to model predictions have red-yellow overlays, while those of lower relevance have blue-green overlays.

The last model evaluated was ShrapML, which resulted in an accuracy across all scan sites of 93.4% (Table 4). Unlike DarkNet-19, no trained model resulted in an instance of 100% positive or negative guesses. However, performance metrics were consistently worse than MobileNetV2. Given the smaller model size of ShrapML, the inference times were much quicker compared to the other models with prediction rates at an average of 3.43 ms per image. GradCAM overlays more closely resembled DarkNet-19 in that many of the heat map intensity points were focused on regions where free fluid was likely to be found or near organs present in the ultrasound scan (Figure 3), except for the HR site. Overall, ShrapML was successful at performing similarly well to these large network structures for this GlobalFAST application, model overfitting was less evident in the results, and overall prediction speed outperformed the other models tested.

**Table 4:** Summary of performance metrics for ShrapML.

Metric	CTS	CTS M-mode	PCS	DH	SR	CC	HR	Average
Accuracy	0.900	0.966	0.908	0.989	0.861	0.965	0.950	0.934
Precision	0.901	0.994	0.917	0.993	0.806	0.967	0.977	0.936
Recall	0.898	0.938	0.897	0.984	0.950	0.963	0.921	0.936
Specificity	0.901	0.994	0.919	0.993	0.772	0.967	0.978	0.932
F1 Score	0.900	0.965	0.907	0.988	0.872	0.965	0.948	0.935
AUROC	0.961	0.998	0.97	0.999	0.928	0.995	0.988	0.977
Inference Time (ms/image)	5.72	3.78	2.63	2.68	3.31	2.83	3.05	3.43

A summary table of average performance metrics for each scan site across all three model architectures is shown in Table 5.

**Table 5:** Summary of performance metrics for each POCUS site.

	CTS	CTS M-Mode	PCS	DH	SR	CC	HR
Accuracy	93.98%	82.11%	94.12%	98.02%	90.61%	88.49%	95.18%
Precision	94.69%	99.42%	96.83%	99.69%	89.00%	86.76%	94.42%
Recall	93.19%	64.60%	91.32%	96.35%	93.52%	95.15%	96.41%
Specificity	94.77%	99.62%	96.92%	99.70%	87.69%	81.82%	93.95%
F1 Score	93.92%	98.11%	93.93%	97.98%	91.04%	90.22%	95.29%
Number of Training Images	23,305	1,652	16,380	11,340	9,455	10,080	9,455

## 4 Discussion

Medical imaging-based triage is critical for both human and veterinary emergency medicine to identify issues early on and ensure resources are properly distributed. In remote or military medicine situations, the lack of skilled personnel makes imaging based-triage less relied upon, but AI prediction models can simplify this for the end user. Here, we focus on the POCUS procedure GlobalFAST®, a widely used triage exam to look for abdominal or thoracic free fluid in injured dogs. The AI models shown in this work can automate predictions for ultrasound results if properly tuned for the application.

Three different AI architectures were evaluated to see which was capable of being trained to distinguish positive injury cases from baseline images. While all models were generally successful at being trained for these applications, strong test performance may not indicate properly trained models. For instance, MobileNetV2 had the highest accuracy, but heat map overlays indicating where the AI was focused were not tracking proper image locations. Model overfit was combatted with the various image augmentation techniques used for the training, but this was insufficient to mimic proper subject variability to create a more robust model for this architecture. This issue was less evident for the other two model architectures, highlighting the importance of AI model selection and validation on ultrasound image applications such as this. However, without more subjects and the variability that those bring, it is hard to fully verify if the developed DarkNet-19 or ShrapML models are suitable. Preliminarily, ShrapML had the strongest performance and prediction rate, making it the most suitable going forward as well as eventual integration for real-time deployment with ultrasound machines.

Focusing on the various scan points in the used POCUS exam, there were obvious differences in the AI model training. Training image sets were not equally sized, but that did not correlate to what scan sites performed the best. The DH site was the overall strongest performing site across all performance metrics. However, this could be due to this scan site having the largest difference between live and cadaveric tissue resulting in a well-trained model. In addition, less augmentation steps were used for this site due to training issues using all affine transformations. More images are needed to address this issue from a wider range of subjects. CTS and HR views also performed well across the three models trained. Worst performing was the M-mode reconstructed chest tube images which could be influenced by the minimal training data used for this model, and thus may be improved with more training data. The CC site was also a lower performing scan site even though more than 10,000 images were used in the training dataset. However, this is mostly influenced by DarkNet-19 having lower performance for this scan site while the other two models had accuracies greater than 96%. Overall, each scan site for this POCUS application was successful as an input for an injury prediction model.

## 5 Conclusion

Artificial intelligence has the potential to simplify triage and injury diagnosis for emergency veterinary medicine. The results shown in this work highlight how AI can be used for automating US detection of intrabdominal and intrathoracic injury detection for veterinary applications. Each scan point reached greater than 80% injury detection accuracy, with most surpassing 90% accuracy. However, more data is still needed to be able to ensure that the AI models are not overfitting the training data and can accurately predict for new subject data. Next steps for this work will expand training datasets so that blind subject testing is possible for confirming generalized models are developed. With more data, these models can be set up for real-time integration with ultrasound devices allowing for early detection of thoracic and abdominal injuries for military working dogs and other canine trauma situations. This will lower the skill threshold for medical imaging-based triage so that these techniques can be more widely used.

## Data availability statement

The datasets presented in this article are not readily available because they have been collected and maintained in a government-controlled database that is located at the US Army Institute of Surgical Research. As such, this data can be made available through the development of a Cooperative Research & Development Agreement (CRADA) with the corresponding author. Requests to access the datasets should be directed to ES, eric.j.snider3.civ@health.mil.

## Ethics statement

The animal study was approved by Research was conducted in compliance with the Animal Welfare Act, the implementing Animal Welfare regulations, and the principles of the Guide for the Care and Use for Laboratory Animals. The Institutional Animal Care and Use Committee at the Department of Defense Military Working Dog Veterinary Services approved all research conducted in this study. The facility where this research was conducted is fully accredited by the AAALAC International. The study was conducted in accordance with the local legislation and institutional requirements.

## Author contributions

SH: Conceptualization, Data curation, Formal analysis, Methodology, Visualization, Writing – original draft, Writing – review & editing. LH: Data curation, Formal analysis, Methodology, Software, Writing – review & editing. TE: Funding acquisition, Writing – review & editing. EV: Writing – original draft, Methodology, Funding acquisition, Data curation, Conceptualization. ES: Conceptualization, Data curation, Formal analysis, Methodology, Writing – original draft, Writing – review & editing.
